# New FLU mutant alleles point to new insights into singlet oxygen‐mediated signal transduction

**DOI:** 10.1111/nph.71499

**Published:** 2026-08-11

**Authors:** Bernhard Grimm

**Affiliations:** ^1^ Humboldt‐Universität zu Berlin, Institute of Biology/Plant Physiology Philippstraße 13 (Building 12) 10115 Berlin Germany

**Keywords:** ^1^O_2_‐mediated signalling, ALA‐biosynthesis, Executer1, FLU, plastid‐derived retrograde signalling, tetrapyrrole biosynthesis

## Abstract

This article is a Commentary on Zhang *et al*. (2026), **252**: 1488–1509.

Over the course of evolution, angiosperms have developed distinct developmental programmes depending on whether they are exposed to light or grown in the dark (known as photomorphogenesis and scotomorphogenesis). Under the influence of light, cellular plastids evolved into the primary energy sources. The light‐dependent processes in the plastids were optimised to the plant's morphology and anatomy for photosynthesis and the associated metabolic pathways. Chlorophyll synthesis, a branch of tetrapyrrole metabolism, also became a light‐dependent process in angiosperms thanks to an enzyme that is active exclusively in the presence of light (Vedalankar & Tripathy, 2019). The unusual light‐dependent protochlorophyllide oxidoreductase (POR), which is only activated by light excitation of protochlorophyllide (Pchlide), the catalytic substrate – which thus also functions as a chromophore – differs structurally, catalytically and regulatorily from the light‐independent POR found in the other photosynthetic organisms.

Light‐dependent Chl synthesis could only be achieved through the development of extremely strict and precise control of metabolite flow in the tetrapyrrole biosynthetic pathway. This was necessary, in order to block the pathway during dark phases, right at the start of the rate‐limiting step of 5‐aminolevulinic acid (ALA) synthesis, since it prevents the accumulation of excessive amounts of photoreactive Pchlide. Light absorption of Pchlide could cause the transfer of the excitation energy to molecular oxygen, thereby forming the highly reactive singlet oxygen (^1^O_2_). Depending on its quantity, ^1^O_2_ essentially triggers two different processes. It either activates a signalling transduction that leads to protective responses or induces cell death.

*…report that analysis of suppressor mutants of the flu/ex1 double mutant has now revealed the identification of new allelic FLU mutants that produce a much more pronounced typical flu phenotype…*

It was rather obvious to the scientists who first analysed plastid‐localized photodynamic processes that the massive disruption caused by reactive oxygen species (ROS) would lead directly to massive membrane damage both inside and outside the chloroplasts, and to the immediate death of the cells and leaf necrotic phenotype. However, it became apparent that in the course of evolutionary adaptation to the negative consequences of light exposure, plants have learnt to defend themselves against the effects of photo‐oxidative stress at an early stage, even in the presence of much more moderate accumulations of ^1^O_2_, by triggering modified gene expression with regulatory molecular responses.

The identification of a tetrapyrrole, ^1^O_2_‐mediated signalling pathway between the plastids and the cell nucleus (retrograde signalling) was, in fact, aided by a fortunate coincidence during the work of Klaus Apel and his group after identification of the *Fluorescent in blue light* (*flu*) mutant 25 years ago. Using a forward‐genetic approach, seedlings grown in the dark (etiolement) were identified in a collection of Arabidopsis ethyl methanesulfonate mutants; these seedlings exhibited high red fluorescence and subsequently died after exposure to light (Meskauskiene *et al*., 2001). The mutated *FLU* gene, encoding a noncanonical tetratricopeptide repeat (TPR) protein, was identified as a negative regulator of Chl biosynthesis. This first posttranslational regulatory protein of Chl synthesis remained one of the important, if not the most important, regulators of the metabolic pathway.

Since FLU could not be detected immunologically by anti‐FLU antibodies in the *flu* mutant, the scientific community was led to believe that *flu* was a knockout mutant. Klaus Apel and his research group primarily used the *flu* mutant to further elucidate the ^1^O_2_‐mediated signal transduction pathway, searching through further genetic experiments for additional components of the pathway that could lead to modified gene expression as a result of the *flu*‐triggered processes. Through second‐site revertants of the *flu* phenotype, the plastid‐localised protein EXECUTER1 was identified using a forward genetics approach (Lee *et al*., 2007). However, the function and role of EXECUTER in preventing ^1^O_2_‐induced cell death remained obscure for a long time. It is only in recent years that initial evidence has emerged to suggest a mechanism by which EXECUTER mediates ^1^O_2_ signalling, transferring information from the plastids to the cytoplasm and nucleus. The oxidation of the conserved tryptophan residue 548 of EXECUTER, induced by ROS, was observed to subsequently trigger proteolytic cleavage of EXECUTER into a shorter peptide, which was proposed to be translocated to the cytoplasm and the nucleus, affecting transcriptional regulation in the nucleus (Dogra *et al*., 2019; Zhao *et al*., 2025).

In an article published in this issue of *New Phytologist*, Zhang *et al*. (2026; pp. 1488–1509) report that analysis of suppressor mutants of the *flu/ex1* double mutant has now revealed the identification of new allelic *FLU* mutants that produce a much more pronounced typical *flu* phenotype – that is, higher PChlide accumulation in etiolated seedlings and thus also likely higher ALA synthesis during the dark phase than the wild‐type *flu* mutant. This phenotype could no longer be repressed by *executer1* (*ex1*) mutations. This work led the authors to hypothesise that the original flu (A262V) mutant is a weak allele and must still exert a weak inhibitory effect on ALA synthesis. The presence of a weak knock‐down allele in the original *flu* mutant was supported by the finding that an FLU (A262V)‐Flag fusion protein is immunologically detectable using the anti‐Flag antibody.

This important work thus demonstrates that a further chapter needs to be added to our understanding of the role of the negative regulator FLU and EXECUTER. It appears that an excess of ^1^O_2_ cannot be regulated by an EXECUTER1‐mediated signalling pathway alone; rather, additional signalling pathways or other secondary messengers may be required to transfer information from the chloroplast to the nucleus.

In this context, attention is now once again being turned both to the initial studies on the destructive photo‐oxidative and photodynamic processes caused by the accumulation of tetrapyrroles. This can be attributed to deregulated tetrapyrrole biosynthesis as well as the more recent work on ^1^O_2_ signalling pathways in plants, which should be understood as a controlled response to tetrapyrrole accumulation. These studies are also physiologically relevant and are enabling a deeper and more novel significance to be realised.

In the early studies of the 1960s, the application of peroxidising herbicides was shown to cause inhibition of protoporphyrinogen oxidase, another enzyme involved in tetrapyrrole biosynthesis that acts before the branching pathway leading to Chl and heme synthesis. The photosensitiser following the application of the herbicides was protoporphyrin, an intermediate product of tetrapyrrole biosynthesis (Matringe *et al*., 1989). Since the 1980s, tetrapyrroles or their precursor ALA have been used in photodynamic therapy against certain forms of cancer (Dougherty *et al*., 1978). Since the 1990s, necrotic cell death resulting from the accumulation of tetrapyrrole metabolites has been demonstrated through the inactivation of many enzymatic steps in porphyrin synthesis in plants (one of the first of numerous studies is by Kruse *et al*., 1995).

Following the publication of the first use of the *flu* mutant, which proved to be a reliable tool for investigating the ^1^O_2_‐mediated signalling pathway due to its ability to accumulate ^1^O_2_ conditionally, further recent studies have analysed mutants of Chl and heme synthesis. The search was on for evidence of further retrograde signalling pathways for the intracellular downstream processes caused by the high photosensitisation of the tetrapyrrole precursors in the chloroplasts. It is now already evident from the new work by Zhang *et al*. (2026) that there is not just one ^1^O_2_ signal pathway generated by excited excess PChlide. It is also noteworthy that there are further ^1^O_2_‐triggered signal pathways generated by other photosensitive tetrapyrroles (Tano *et al*., 2023). I find the studies involving an *fc2* mutant (which encodes Ferrochelatase 2, an isoform of the final enzyme in heme synthesis) particularly noteworthy, as this mutant also conditionally accumulates ^1^O_2_ (Alamdari *et al*., 2021; Rai *et al*., 2025). However, there is still a long way to go to identify the factors involved in these signalling pathways that also act as secondary messengers to relay the information to the cytoplasm and the cellular nucleus.

The Apel research group had primarily devoted its studies to elucidating the molecular processes underlying the ^1^O_2_ signalling pathways triggered by the *flu* mutation. However, following the discovery of the FLU protein, numerous questions remained regarding the posttranslational inactivation of ALA synthesis by this negative regulator; this will need to be addressed in the future. Initially, a crucial clue came from Lin Liu's research group, who, using the soluble portion of the FLU protein – the TPR domain – a dimer of glutamyl‐tRNA reductase (GluTR), the first enzyme in tetrapyrrole biosynthesis, and the GluTR‐binding protein, elucidated its structure through modelling and crystallography (Fang *et al*., 2016). The group proposed that the binding of a dimer of the FLU‐TPR domain to the C‐terminal ends of the GluTR dimer prevents the binding of glutamyl‐tRNA_(Glu)_, the substrate of GluTR (Zhang *et al*., 2015). Confirmation of this idea is still pending.

It also remains unclear how the FLU protein, which is bound to the thylakoid membrane and is also associated with POR and other proteins involved in the late stages of Chl biosynthesis, manages to pull GluTR to the thylakoid membrane for inactivation when required. Furthermore, FLU not only inactivates ALA synthesis at night but is also involved in the fine‐tuning of ALA synthesis during the day to balance the supply of ALA precursor molecules required for the production of tetrapyrrole end products (Hou *et al*., 2021). This raises the pressing question of which signals or mechanisms for downregulating ALA synthesis trigger this inactivation process during the day. Does it correspond to the FLU‐mediated control mechanism at night?

We must note that the original *flu* mutant, which contributed to much subsequent work on ^1^O_2_ signalling or the posttranslational regulation of ALA synthesis in higher plants, was only identified after more than two decades as a knock‐down mutant, which at the time led to the fortunate coincidence that the suppression mutant *ex1*, in turn, revealed the surprising finding of the reversal of the suppression triggered by gene expression. It therefore remains important to acknowledge that even exciting earlier results must continue to face definitive clarification in later studies using perhaps more sophisticated methods, and that the original findings may be **relativised**. It therefore remains exciting to learn more about the future interconnections of regulatory processes for countering and mitigating ^1^O_2_‐induced stress in chloroplasts.

## Disclaimer

The New Phytologist Foundation remains neutral with regard to jurisdictional claims in maps and in any institutional affiliations.
